# Supplementary material for: BRCA2 antagonizes classical and alternative nonhomologous end-joining to prevent gross genomic instability
Source: Nat Commun. 2017 Nov 13;8:1470. doi: 10.1038/s41467-017-01759-y (PMC5684403; doi:10.1038/s41467-017-01759-y)
Supplement: Supplementary file 1 — Supplementary Information [file 41467_2017_1759_MOESM1_ESM.pdf]

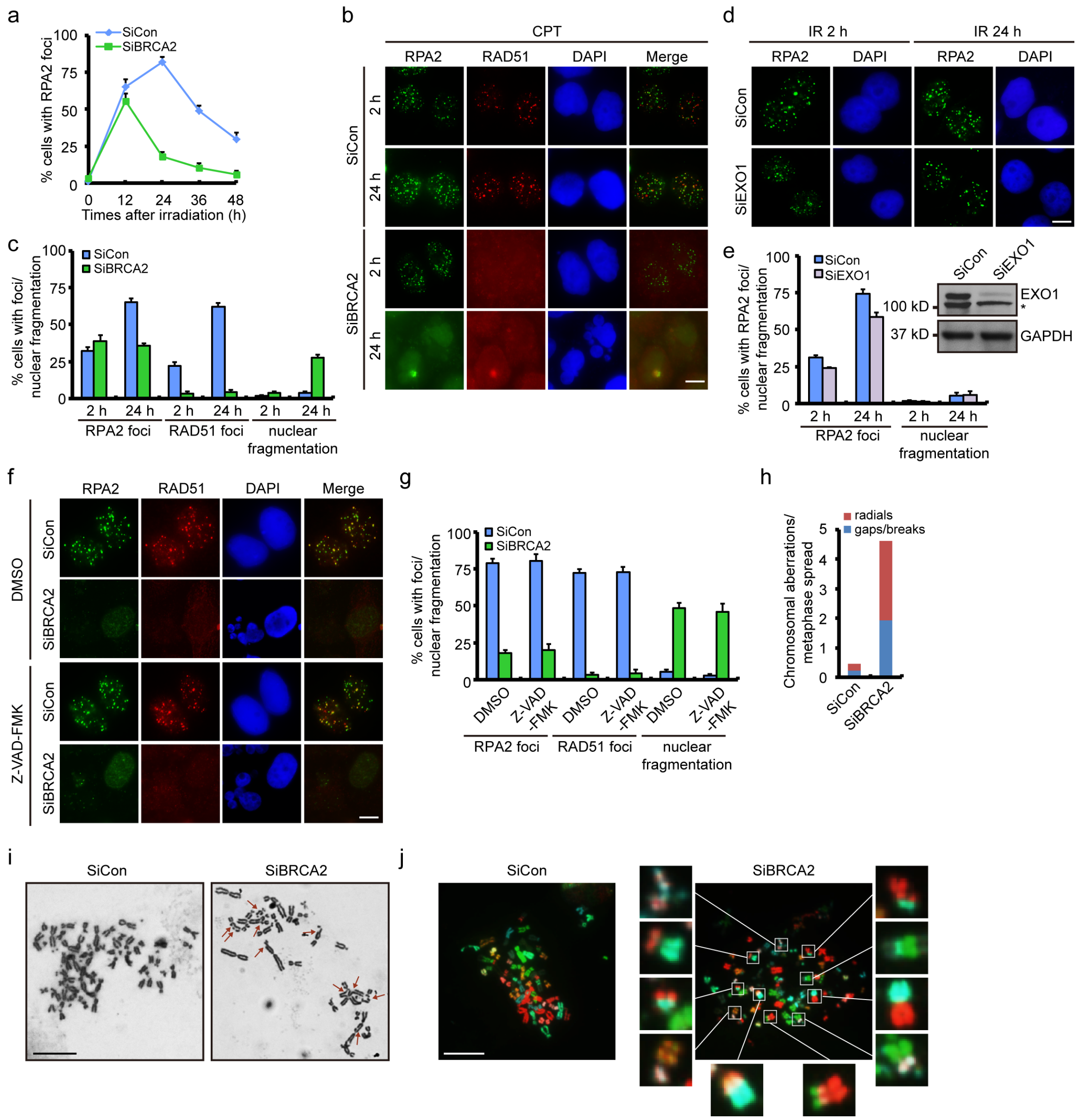

Supplementary Figure 1. Inactivation of BRCA2 accelerates the dissolution of RPA2 foci and causes massive nuclear fragmentation. (**a**) HeLa cells transfected with indicated siRNAs were exposed to 10 Gy IR and then allowed to recover for the indicated time periods before being processed for immunofluorescence using antibody against RPA2. Data represent mean ± SEM of three independent experiments. (**b**) HeLa cells transfected with indicated siRNAs were treated with 0.5 μM CPT for 0.5 hour. After washing, cells were shifted to fresh medium and allowed to recover for 2 hr or 24 hr before being processed for immunofluorescence using antibodies against RPA2 and RAD51.

Representative RPA2/RAD51 foci and DAPI-stained nuclei are shown. (**c**) Quantification of RPA2/RAD51 foci and nuclear fragmentation. Data represent mean ± SEM of three independent experiments. (**d**) EXO1 depletion has a minimal effect on the kinetics of RPA2 foci disappearance and nuclear integrity. HeLa cells transfected with indicated siRNAs were exposed to 10 Gy IR and then allowed to recover for 2 hr or 24 hr before being processed for immunofluorescence using antibody against RPA2. (**e**) Quantification of RPA2 foci and nuclear fragmentation. Data represent mean ± SEM of three independent experiments. Knockdown efficiency was confirmed by Western blotting. The asterisk indicates a nonspecific band. (**f**) HeLa cells transfected with indicated siRNAs were exposed to 10 Gy IR. 6 hr later, cells were treated with 20 μM Z-VAD-FMK or DMSO for 18 hr and then processed for immunofluorescence using indicated antibodies. (**g**) Quantification of RPA2/RAD51 foci and nuclear fragmentation. Data represent mean ± SEM of three independent experiments. (**h and i**) HeLa cells transfected with indicated siRNAs were exposed to 10 Gy IR and then allowed to recover for 18 hr. Metaphase spreads were then prepared according to standard protocols. Chromosomal aberrations are marked by red arrows (**i**). Quantification of chromosomal aberrations in indicated cells after IR treatment is shown (**h**). More than 50 metaphases were examined for each experiment. (**j**) Multicolor fluorescence in situ hybridization (M-FISH) analysis of control and BRCA2-depleted HeLa cells are showed. Metaphase spreads of IR treated cells were hybridized with M-FISH probes. Scale bars, 10 μm.

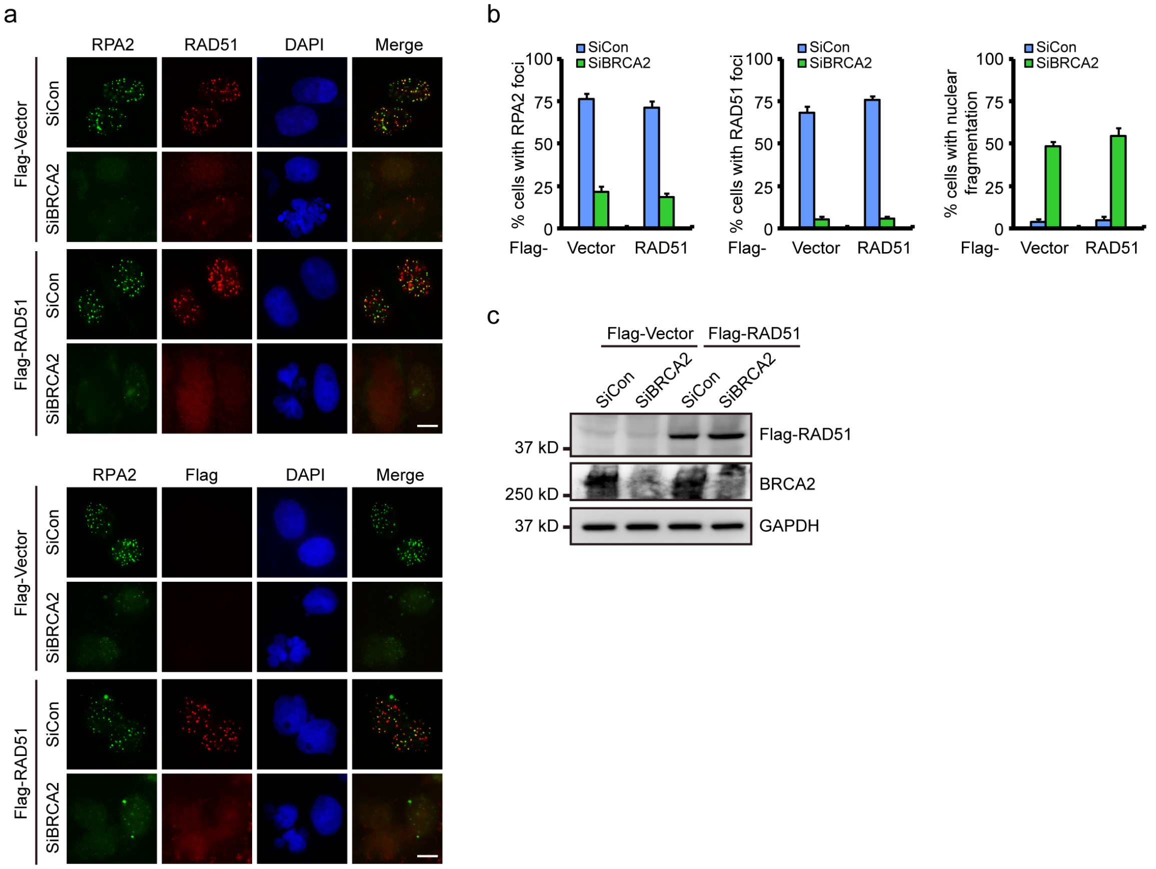

Supplementary Figure 2. BRCA2 suppresses gross genomic instability independently of RAD51. (a) HeLa cells stably expressing Flag-tagged RAD51 were transfected with siRNA against BRCA2. 48 hr posttransfection, cells were exposed to 10 Gy IR and then allowed to recover for 24 hr before being processed for immunofluorescence using antibodies against RPA2 and RAD51 or the Flag tag. Representative RPA2/RAD51 foci and DAPI-stained nuclei are shown. (**b**) Quantification of RPA2/RAD51 foci and nuclear fragmentation. Data represent mean ± SEM of three independent experiments. Over 100 cells were counted in each experiment. (**c**) RAD51 expression was confirmed by Western blotting. Scale bars, 10 μm.

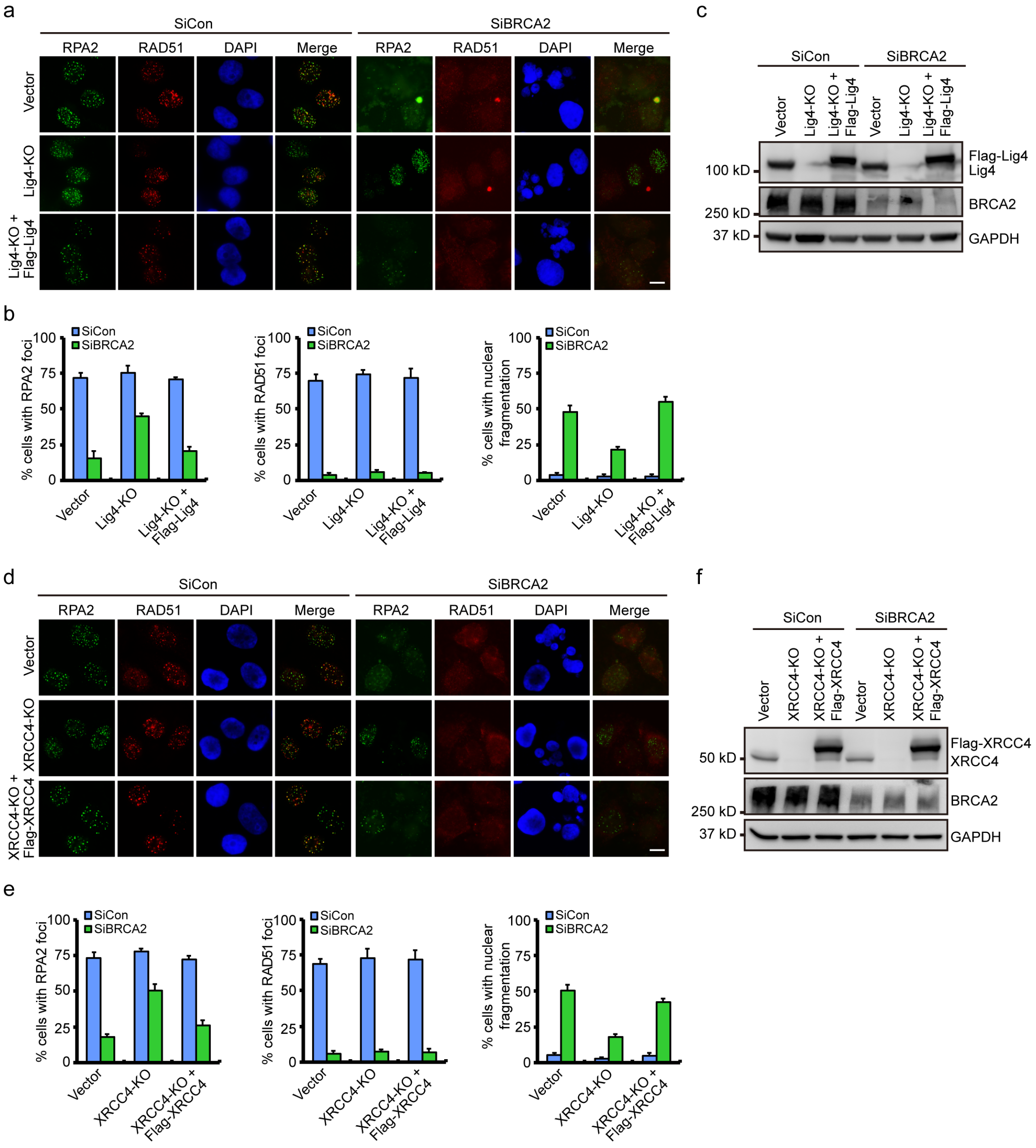

Supplementary Figure 3. C-NHEJ is partially responsible for gross genomic instability in BRCA2-depleted cells. (**a**) Lig4-deficient HeLa cells stably expressing wild-type Lig4 were transfected with control siRNA or siRNA against BRCA2. 48 hr posttransfection, cells were exposed to 10 Gy IR and then allowed to recover for 24 hr before being processed for immunofluorescence using antibodies against RPA2 and RAD51. Representative RPA2/RAD51 foci and DAPI-stained nuclei are shown. (**b**)

Quantification of RPA2/RAD51 foci and nuclear fragmentation. Data represent mean ± SEM of three independent experiments. Over 100 cells were counted in each experiment. (**c**) The expression of Lig4 was confirmed by Western blotting. (**d**) XRCC4-deficient HeLa cells stably expressing wild-type XRCC4 were transfected with control siRNA or siRNA against BRCA2. 48 hr posttransfection, cells were exposed to 10 Gy IR and then allowed to recover for 24 hr before being processed for immunofluorescence using antibodies against RPA2 and RAD51. Representative RPA2/RAD51 foci and DAPI-stained nuclei are shown. (**e**) Quantification of RPA2/RAD51 foci and nuclear fragmentation. Data represent mean ± SEM of three independent experiments. Over 100 cells were counted in each experiment. (**f**) The expression of XRCC4 was confirmed by Western blotting. Scale bars, 10 μm.

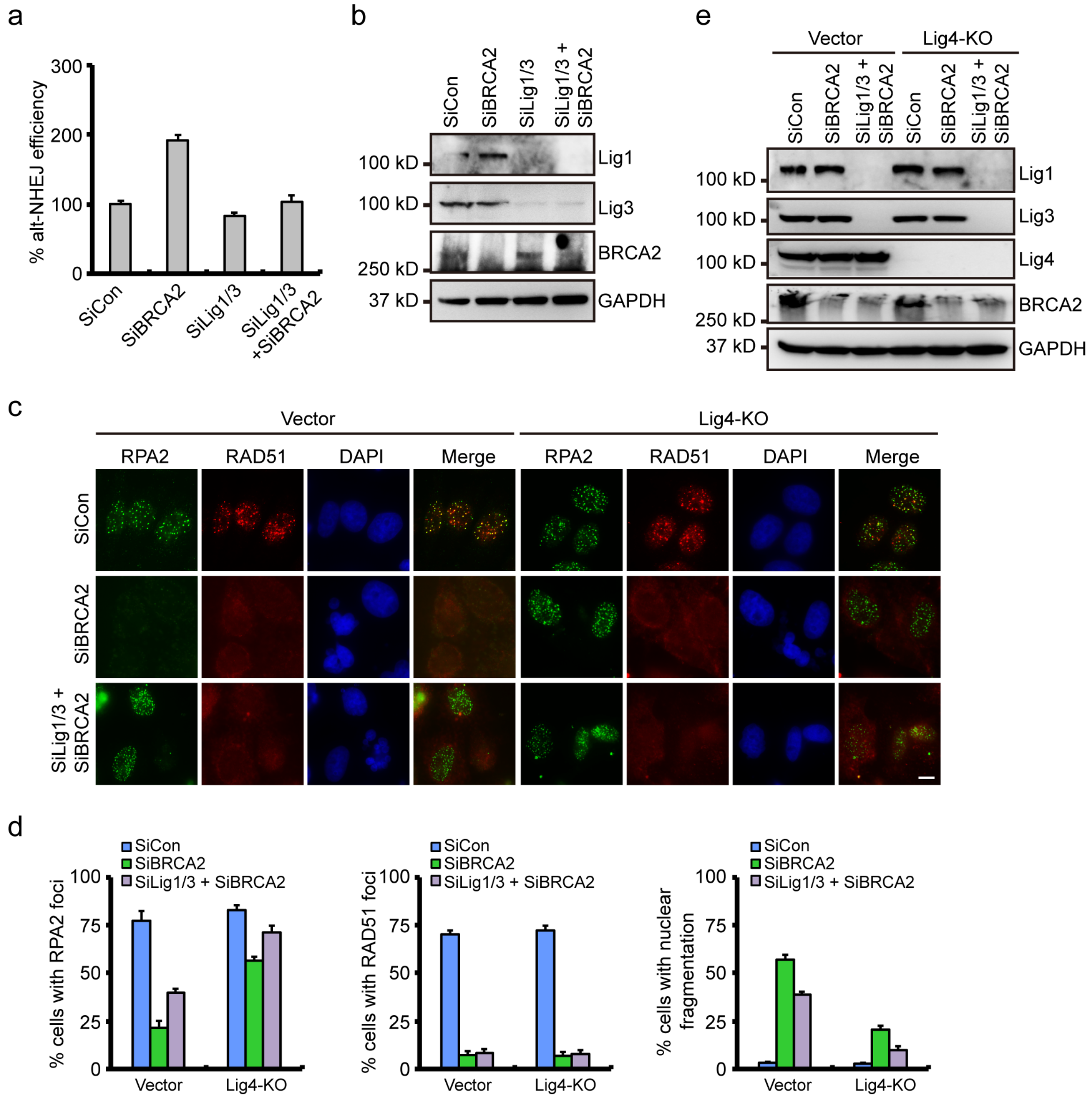

Supplementary Figure 4. Alt-NHEJ is partially responsible for gross genomic instability in BRCA2-depleted cells. (**a**) U2OS alt-NHEJ-EGFP cells were transfected with indicated siRNAs. 24 hr posttransfection, cells were electroporated with pCBASce plasmid. After 48 hr, the percentage of GFP-positive cells was determined by FACS. Results represent mean ± SEM of three independent experiments. (**b**) Knockdown efficiency was confirmed by Western blotting. (**c**) Wild-type or Lig4-deficient HeLa cells were transfected with indicated siRNAs. 48 hr posttransfection, cells were exposed to 10 Gy IR and then allowed to recover for 24 hr before being processed for immunofluorescence using antibodies against RPA2 and RAD51. Representative

RPA2/RAD51 foci and DAPI-stained nuclei are shown. (**d**) Quantification of RPA2/RAD51 foci and nuclear fragmentation. Data represent mean ± SEM of three independent experiments. Over 100 cells were counted in each experiment. (**e**) Knockdown efficiency was confirmed by Western blotting. Scale bar, 10 μm.

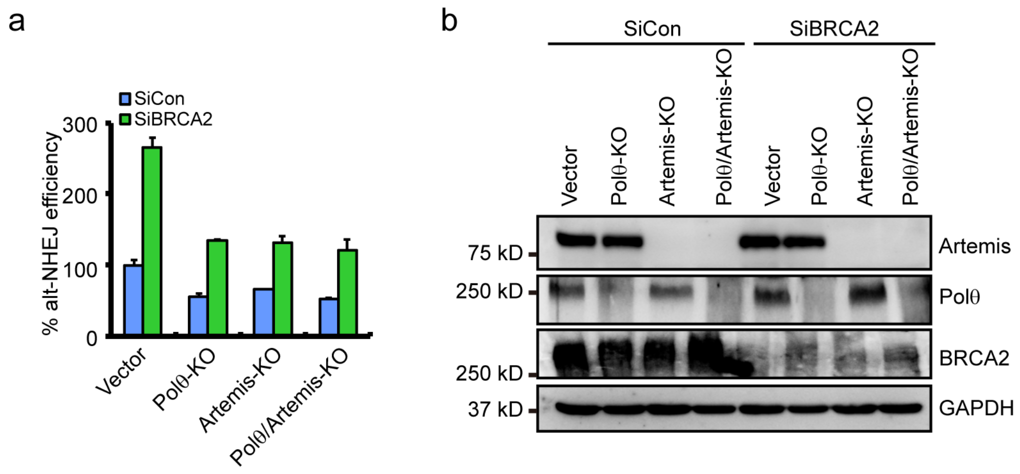

Supplementary Figure 5. Artemis and Polθ function in the same pathway to promote alt-NHEJ. (**a**) Wild-type, Artemis-, Polθ-, or Artemis/Polθ-deficient U2OS alt-NHEJ-EGFP cells were transfected with the control siRNA or siRNA against BRCA2. 24 hr later, cells were electroporated with pCBASce plasmid. After 48 hr, the percentage of GFP-positive cells was determined by FACS. Results represent mean ± SEM of three independent experiments. (**b**) Knockout/knockdown efficiency was confirmed by Western blotting.

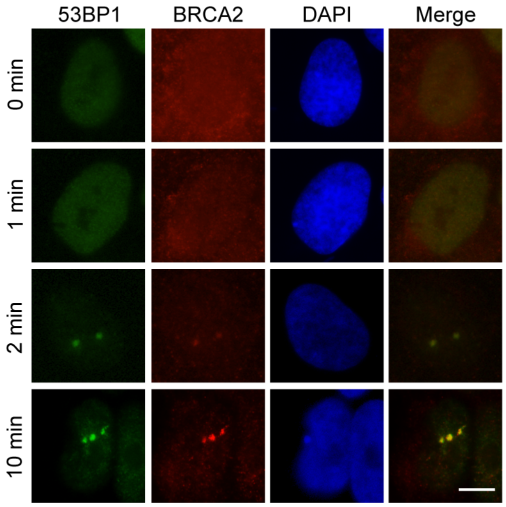

Supplementary Figure 6. BRCA2 is rapidly recruited to sites of DNA damage. HeLa cells were micro-irradiated and then allowed to recover for the indicated times before being processed for immunofluorescence using antibodies against 53BP1 and BRCA2. Representative images taken at the indicated times after laser microirradiation are shown. Scale bar, 10 μm.

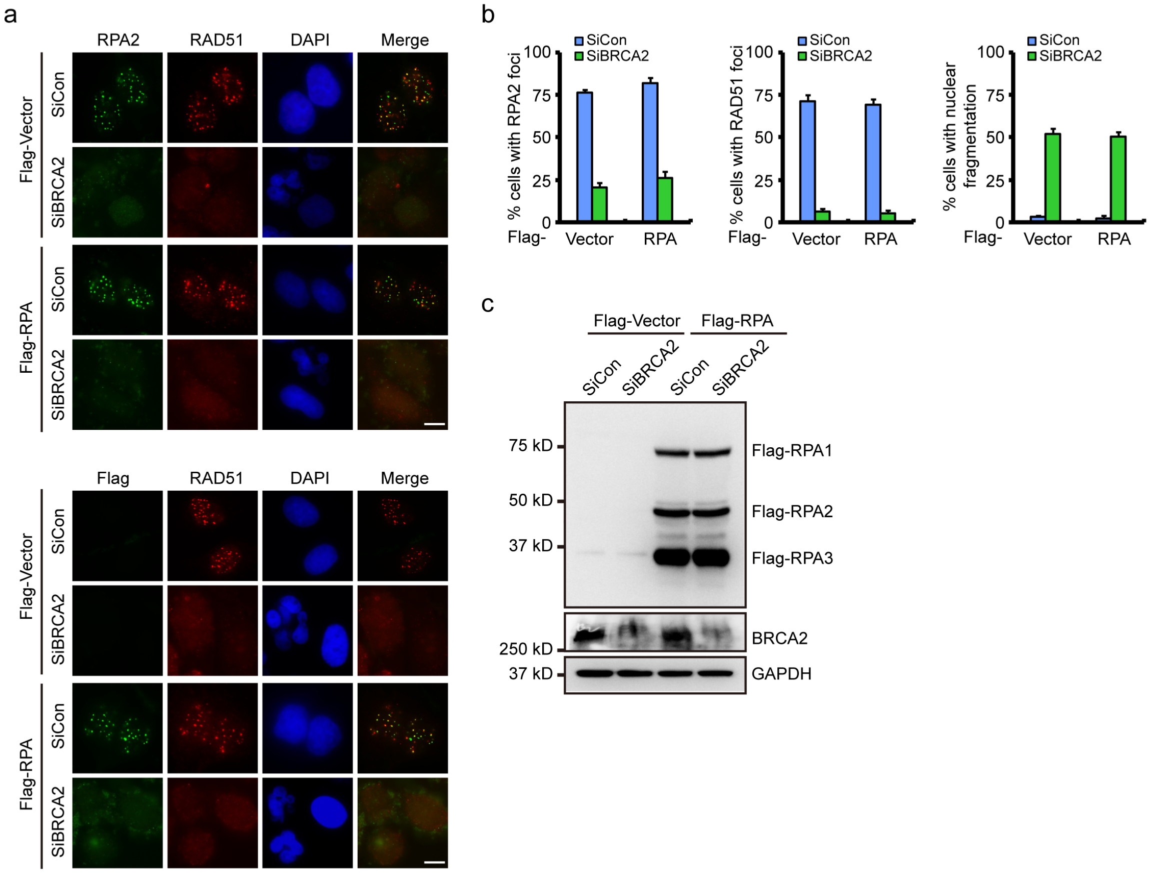

Supplementary Figure 7. Overexpression of RPA is unable to reverse the defects caused by BRCA2 depletion. (a) BRCA2-depleted HeLa cells were electroporated with plasmids encoding Flag-tagged RPA1, RPA2 and RPA3. 24 hr later, cells were exposed to 10 Gy IR and then allowed to recover for 24 hr before being processed for immunofluorescence using antibodies against RAD51 and RPA2 or the Flag tag. Representative RPA2/RAD51 foci and DAPI-stained nuclei are shown. (**b**) Quantification of RPA2/RAD51 foci and nuclear fragmentation. Data represent mean ± SEM of three independent experiments. Over 100 cells were counted in each experiment. (**c**) RPA expression was confirmed by Western blotting. Scale bars, 10 μm.

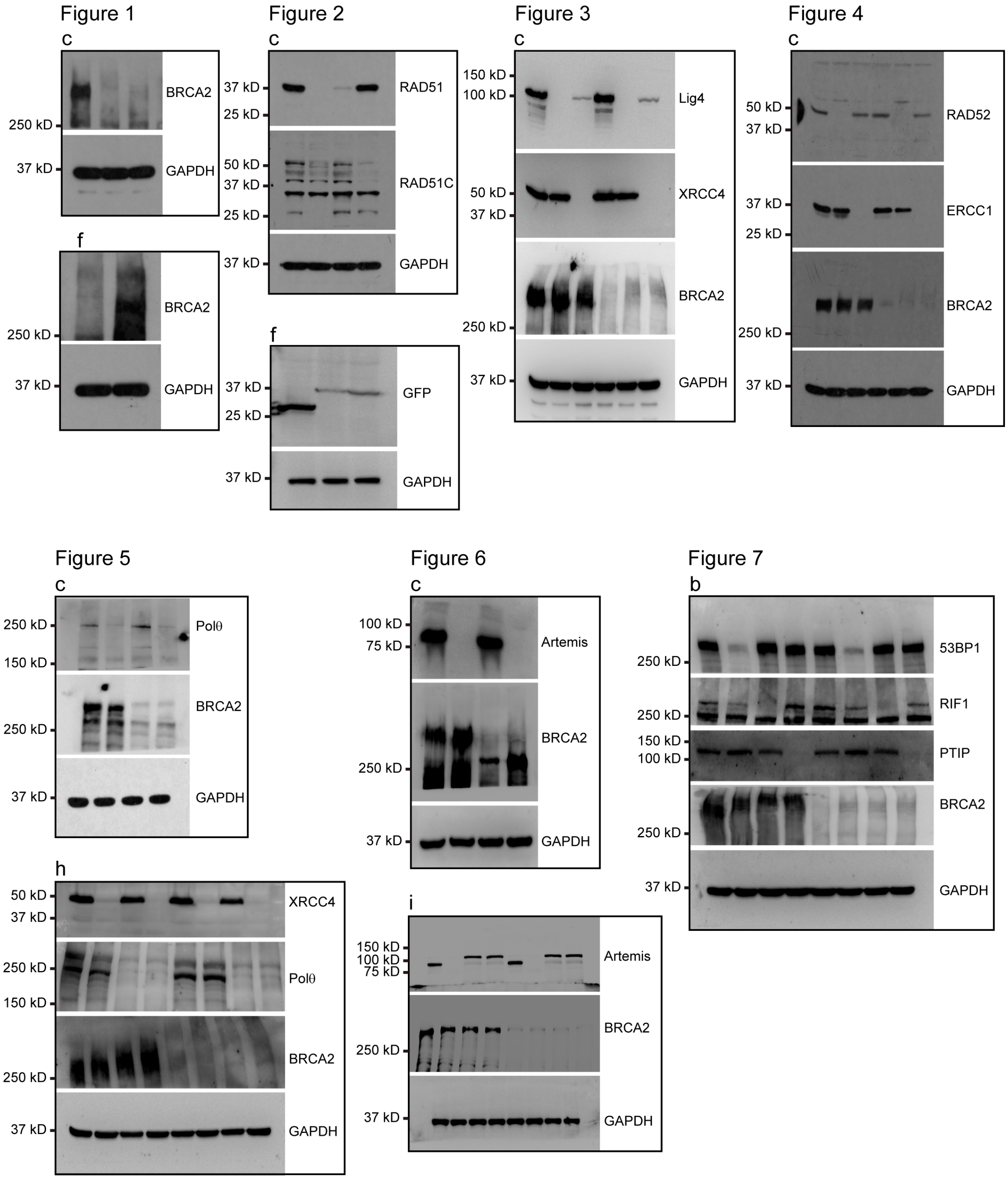

Supplementary Figure 8. Uncropped immunoblots.
