# Supplementary material for: BRCA2 antagonizes classical and alternative nonhomologous end-joining to prevent gross genomic instability
Source: Nat Commun. 2017 Nov 13;8:1470. doi: 10.1038/s41467-017-01759-y (PMC5684403; doi:10.1038/s41467-017-01759-y)
Supplement: Supplementary file 3 — Description of Additional Supplementary Files [file 41467_2017_1759_MOESM3_ESM.pdf]

Description of Additional Supplementary Files

File Name: Supplementary Movie 1
Description: Nuclear fragmentation occurs as a result of chromosome missegregation in BRCA2-depleted cells. HeLa cells stably expressing GFP-tagged histone H2B (H2B-GFP) were transfected with control siRNA. After 48 hr, cells were exposed to 10 Gy IR and allowed to recover for 18 hr. Time-lapse live cell imaging was immediately carried out and images were captured every 5 min for 6 hr. The movies represent a time span of 6 hr (from 18 hr to 24 hr after IR treatment).

File Name: Supplementary Movie 2
Description: Nuclear fragmentation occurs as a result of chromosome missegregation in BRCA2-depleted cells. HeLa cells stably expressing GFP-tagged histone H2B (H2B-GFP) were transfected with siRNA against BRCA2. After 48 hr, cells were exposed to 10 Gy IR and allowed to recover for 18 hr. Time-lapse live cell imaging was immediately carried out and images were captured every 5 min for 6 hr. The movies represent a time span of 6 hr (from 18 hr to 24 hr after IR treatment).
